# Self Inflicted Injuries among Children in United States – Estimates from a Nationwide Emergency Department Sample

**DOI:** 10.1371/journal.pone.0069874

**Published:** 2013-07-18

**Authors:** Naseem Sulyman, Min Kyeong Kim, Sankeerth Rampa, Veerasathpurush Allareddy, Romesh P. Nalliah, Veerajalandhar Allareddy

**Affiliations:** 1 Department of Pediatric Critical Care, Rainbow Babies and Children’s Hospital, University Hospitals, Case Western Reserve University, Cleveland, Ohio, United States of America; 2 Harvard School of Dental Medicine, Boston, Massachusetts, United States of America; 3 School of Rural Public Health, Texas A & M University, College Station, Texas, United States of America; 4 Department of Developmental Biology, Harvard University, Boston, Massachusetts, United States of America; Old Dominion University, United States of America

## Abstract

**Objective:**

The objectives of the current study are to provide nationally representative estimates of hospital based emergency department visits (ED) attributed to self inflicted injuries and attempted suicides among children in United States; and to identify potential methods of such intentional self inflicted injuries and attempted suicides.

**Methods:**

The Nationwide Emergency Department Sample (year 2007) was used. All ED visits occurring among children (aged ≤18 years) with an External Cause of Injury for any of self inflicted injuries were selected. Outcomes examined include hospital ED charges and hospitalization charges. All estimates were projected to national levels.

**Results:**

77,420 visits to hospital based emergency departments were attributed to self inflicted injuries among children (26,045 males and 51,370 females). The average age of the ED visits was 15.7 years. 134 patients died in ED’s (106 males and 28 females) and 93 died in hospitals following in-patient admission (75 males and 18 females). A greater proportion of male ED visits were discharged routinely as opposed to female ED visits (51.1% versus 44%). A greater proportion of male ED visits also died in the emergency departments compared to female visits (0.4% versus 0.05%). 17,965 ED visits necessitated admission into same hospital. The mean charge for each ED visit was $1,874. Self inflicted injuries by poisoning were the most frequently reported sources accounting for close to 70% of all ED visits.

**Conclusions:**

Females comprise a greater proportion of ED visits attributed to self inflicted injuries. 227 children died either in the ED’s or in hospitals. The current study results highlight the burden associated with such injuries among children.

## Introduction

Suicide is one of the leading causes of death among children and adolescents [1]. Suicide or deaths resulting from self inflicted injuries is estimated to be 0.8 per 100,000 population among children aged between 10 and 14 years [1]. Even though hospitalizations resulting from self inflicted injuries among children have been reported to be decreasing during the past few decades, this is still a major public health concern in the United States [2]. Nationwide estimates from previous decades using nationally representative samples such as the National Hospital Ambulatory Medical Care Survey indicate that the annual rate of emergency visits attributed to self-harm was 225.3 per 100,000 populations among youth [3]. Self-inflicted injuries are relatively common, serious, and are frequently reported among adolescents and young adults [4]. Self-inflicted injuries and suicide are both preventable; therefore, it is imperative to examine the characteristics of young populations visiting the emergency department for such reasons. For instance, many studies have described that youths with pediatric bipolar disorder (PBD) are at a greater risk of suicide than respective average youths [5], [6]. Insights into a category of children and adolescents who may be at a higher risk for self-inflicted injuries and suicidal attempts will facilitate in constructing a more focused approach in delivering effective treatments to avoid such unfavorable outcome. The objectives of the current study are to provide nationally representative estimates of hospital based emergency department (ED) visits attributed to self inflicted injuries and attempted suicides among children in the United States; and to identify potential methods of such intentional self inflicted injuries and attempted suicides.

## Materials and Methods

The Nationwide Emergency Department Sample (NEDS) for the year 2007 of the Healthcare Cost and Utilization Project (HCUP) was used for the current study. The NEDS is the largest all-payor hospital based emergency department database. It is a 20% stratified sample of all hospital based emergency department (ED) visits in the United States and draws its sample from 27 participating states [7].

Per University Hospitals, Case Medical Center, Institutional review board and in agreement with Federal Regulations 45 CFR 46.101 (b) “Research involving the collection or study of existing data, documents, records, pathological specimens, or diagnostic specimens, if these sources are **publicly available** or if the information is recorded by the investigator in such a manner that **subjects cannot be identified**, directly or through identifiers linked to the subjects” such studies are permitted to be classified as research that is “exempt” from IRB full or expedited review. IRB was not consulted for approval since the current study was a retrospective analysis of hospitals based discharge dataset that is available publicly for purchase from Agency for Healthcare Research and Quality (AHRQ). The senior author (VA) completed the data user agreement with HCUP-AHRQ and the obtained data. The HCUP-AHRQ data user agreement precludes reporting individual cell counts ≤10 to preserve patient confidentiality. Consequently, these numbers were not reported in the current study.

All ED visits occurring among children (aged ≤18 years) with an External Cause of Injury (E-Code) for any of the self inflicted injuries were selected for analysis. Characteristics of all ED visits including age, gender, insurance status, disposition of patient from emergency department, disposition of patient following in-patient admission into the same hospital, median household income based on zip code of patient resident location, teaching status of hospital, and geographic region were examined. Emergency department charges and hospitalization charges were also examined. Each ED visit in the NEDS database has an assigned sample weight and this was used to project all estimates to national levels. Descriptive statistics were used to summarize the data. For all analyses, the NEDS hospital stratum was the stratification unit and each individual ED visit was the unit of analysis. All statistical analyses were conducted using SAS (version 9.2).

## Results

During the year 2007, a total of 77,420 visits to hospital based emergency departments were attributed to self inflicted injuries among children aged ≤18 years. Of these, 26,045 visits occurred among males while females accounted for 51,370 visits (Table 1). Information regarding gender was not available for 5 visits. The average age of the ED visits was 15.7 years. About 75.5% of all ED visits occurred during weekdays. Close to 46.4% of ED visits were discharged routinely from the emergency departments, while 8.4% were transferred to another short term hospital, 18.9% were transferred to other facilities (including including skilled nursing facility, intermediate care, and another type of facility), 0.2% to home health care, 0.3% were discharged from the emergency departments against medical advice, 23.2% were admitted as inpatients into the same hospital, and for 2.4% the disposition information was not known. 134 patients died in the emergency departments (106 males and 28 females). Private insurance plans were the major listed payors (49%) for all ED visits. A greater proportion of male ED visits were discharged routinely as opposed to female ED visits (51.1% versus 44%). A greater proportion of male ED visits also died in the emergency departments compared to female visits (0.4% versus 0.05%).

**Table 1:** Characteristics of Hospital Based Emergency Department Visits with Suicide and Intentional Self Inflicted Injuries Among Children.

Characteristic	Response	All ED Visits	Male	Female
		(N = 77420)	(N = 26045)	(N = 51370)
Admission timing	Admission on Monday- Friday	57654 (75.5%)	19266 (74%)	38383 (74.7%)
	Admission on Saturday-Sunday	19766 (24.5%)	6779 (26%)	12987 (25.3%)
Disposition of patient from the ED	Routine discharge	35903 (46.4%)	13308 (51.1%)	22595 (44%)
	Transfer to short term facility	6490 (8.4%)	1918 (7.4%)	4572 (8.9%)
	Other transfers, including skilled nursing facility, intermediate care, and anothertype of facility	14628 (18.9%)	4337 (16.6%)	10286 (20%)
	Home health care	182 (0.2%)	67 (0.3%)	116 (0.2%)
	Against medical advice	265 (0.3%)	108 (0.4%)	157 (0.3%)
	Admitted as an inpatient to this hospital	17965 (23.2%)	5681 (21.8%)	12283 (23.9%)
	Died in ED	134 (0.2%)	106 (0.4%)	28 (0.05%)
	Not admitted, destination unknown	1854 (2.4%)	520 (2%)	1333 (2.6%)
Median household income quartiles for patient’s ZIP Code	$1–$37,999	19081 (25.2%)	6761 (26.6%)	12321 (24.4%)
	$38,000–$46,999	20738 (27.3%)	6790 (26.7%)	13948 (27.7%)
	$47,000–$61,999	19812 (26.1%)	6670 (26.2%)	13142 (26.1%)
	$62,000 or more	16181 (21.3%)	5197 (20.4%)	10980 (21.8%)
Insurance status	Medicare	259 (0.3%)	137 (0.5%)	122 (0.2%)
	Medicaid	26472 (34.4%)	9040 (34.9%)	17432 (34.1%)
	Private including HMO	37704 (49%)	12221 (47.1%)	25478 (49.9%)
	Uninsured	9410 (12.2%)	3400 (13.1%)	6010 (11.8%)
	Other	3133 (4.1%)	1122 (4.3%)	2011 (3.9%)
Hospital region	Northeast	15175 (19.6%)	5278 (20.3%)	9898 (19.3%)
	Midwest	21527 (27.8%)	6958 (26.7%)	14564 (28.3%)
	South	24666 (31.9%)	8664 (33.3%)	16002 (31.1%)
	West	16051 (20.7%)	5145 (19.7%)	10906 (21.2%)
Hospital location and teaching status	Metropolitan non teaching	34292 (44.3%)	11172 (42.9%)	23115 (45%)
	Metropolitan teaching	28504 (36.8%)	9763 (37.5%)	18741 (36.5%)
	Non- metropolitan	14624 (18.9%)	5111 (19.6%)	9513 (18.5%)

Note: Individual cell counts may not add to the global total because of missing values.

A total of 17,965 ED visits (23.3% of the total visits) necessitated admission into the same hospital following an ED visit (21.8% in males versus 23.9% in females). Among those ED visits requiring inpatient admission into the same hospital, 59.3% were discharged routinely (57.8% in males versus 60% in females), while 6.9% were transferred to another short term hospital (6.3% in males versus 7.1% in females), 31.6% were transferred to other facilities (32.6% in males versus 31.1% in females), 0.4% to home health care (0.6% in males versus 0.3% in females), 0.9% were discharged against medical advice (0.9% in males versus 0.9% in females), and for 0.4% the destination was unknown (0.4% in males versus 0.4% in females). 93 children died in the hospitals (75 males and 18 females).

The mean charge for each ED visit was $1,874 (std. error is 47.06). The total ED charges for the entire United States were $113.16 million. The ED charges information were missing for close to 21% of all visits. The mean charges (including ED charge and inpatient charges) for those ED visits which required in-patient admission into the same hospital was $12,801 (std. error is 588.81). The total US hospitalization charges was $ 227.85 million.

The sources for self inflicted injuries are summarized in Table 2. Self inflicted injuries by poisoning were the most frequently reported sources accounting for close to 70% of all ED visits. Other four major sources of self inflicted injuries among all ED visits were injuries by cutting and piercing instruments (27.4% of all ED visits, 24% in males versus 29.2% in females); poisoning by analgesics, antipyretics, or antirheumatics (25.9% of all ED visits, 16.5% in males versus 30.7% in females); poisoning by tranquilizers and other psychotropic agents (17.3% of all ED visits, 16.8% in males versus 17.5% in females); and poisoning by other specified drugs and medicinal substances (16.3% of all ED visits, 15.4% in males versus 16.7% in females).

**Table 2:** Source of Suicide and Intentional Self Inflicted Injuries Reported.

Source of Injury (ICD-9-CM Code)	All ED Visits	Male	Female
	(N = 77420)	(N = 26045)	(N = 51370)
Suicide and self-inflicted poisoning by Analgesics, antipyretics, and antirheumatics (E950.0)	20077 (25.9%)	4298 (16.5%)	15779 (30.7%)
Suicide and self-inflicted poisoning by Barbiturates (950.1)	121 (0.2%)	47 (0.2%)	74 (0.1%)
Suicide and self-inflicted poisoning by Other sedatives and hypnotics (950.2)	1400 (1.8%)	569 (2.2%)	831 (1.6%)
Suicide and self-inflicted poisoning by Tranquilizers and other psychotropic agents (950.3)	13390 (17.3%)	4389 (16.8%)	9002 (17.5%)
Suicide and self-inflicted poisoning by Other specified drugs and medicinal substances (950.4)	12611 (16.3%)	4013 (15.4%)	8598 (16.7%)
Suicide and self-inflicted poisoning by Unspecified drug or medicinal substance (950.5)	2084 (2.7%)	604 (2.3%)	1479 (2.9%)
Suicide and self-inflicted poisoning by Agricultural and horticultural chemical and pharmaceutical preparations (950.6)	103 (0.1%)	52 (0.2%)	50 (0.1%)
Suicide and self-inflicted poisoning by Corrosive and caustic substances (950.7)	541 (0.7%)	180 (0.7%)	360 (0.7%)
Suicide and self-inflicted poisoning by Arsenic and its compounds (950.8)	22 (0.03%)	DS	DS
Suicide and self-inflicted poisoning by Other and unspecified solid and liquid substances (950.9)	2230 (2.9%)	930 (3.6%)	1300 (2.5%)
Suicide and self-inflicted poisoning by Gas distributed by pipeline (E951.0)	DS	DS	DS
Suicide and self-inflicted poisoning by Liquefied petroleum gas distributed in mobile containers (E951.1)	DS	DS	DS
Suicide and self-inflicted poisoning by Other utility gas (E951.8)	DS	DS	DS
Suicide and self-inflicted poisoning by Motor vehicle exhaust gas (952.0)	42 (0.05%)	DS	DS
Suicide and self-inflicted poisoning by Other carbon monoxide (952.1)	15 (0.02%)	15 (0.06%)	-
Suicide and self-inflicted poisoning by Other specified gases and vapors (952.8)	133 (0.2%)	87 (0.3%)	46 (0.1%)
Suicide and self-inflicted poisoning by Unspecified gases and vapors (952.9)	31 (0.04%)	DS	DS
Suicide and self-inflicted injury by Hanging (E953.0)	1475 (1.9%)	999 (3.8%)	476 (0.9%)
Suicide and self-inflicted injury by Suffocation by plastic bag (E953.1)	23 (0.03%)	11 (0.04%)	11 (0.02%)
Suicide and self-inflicted injury by Other specified means (E953.8)	357 (0.5%)	191 (0.7%)	166 (0.3%)
Suicide and self-inflicted injury by Unspecified means (E953.9)	58 (0.07%)	31 (0.1%)	27 (0.05%)
Suicide and self-inflicted injury by submersion [drowning] (E954)	60 (0.07%)	17 (0.07%)	43 (0.08%)
Suicide and self-inflicted injury by Handgun (E955.0)	95 (0.1%)	83 (0.3%)	12 (0.02%)
Suicide and self-inflicted injury by Shotgun (E955.1)	26 (0.03%)	26 (0.1%)	-
Suicide and self-inflicted injury by Hunting rifle (E955.2)	53 (0.07%)	39 (0.1%)	13 (0.03%)
Suicide and self-inflicted injury by Military firearms (E955.3)	DS	DS	DS
Suicide and self-inflicted injury by Other and unspecified firearm (E955.4)	81 (0.1%)	81 (0.3%)	-
Suicide and self-inflicted injury by Explosives (E955.5)	DS	DS	DS
Suicide and self-inflicted injury by Air gun (E955.6)	141 (0.2%)	128 (0.5%)	13 (0.02%)
Suicide and self-inflicted injury by Paintball gun (E955.7)	DS	DS	DS
Suicide and self-inflicted injury by Unspecified firearms (E955.9)	17 (0.02%)	DS	DS
Suicide and self-inflicted injury by cutting and piercing instrument (E956)	21245 (27.4%)	6246 (24%)	15000 (29.2%)
Suicide and self-inflicted injuries by jumping from high place - Residential premises (E957.0)	135 (0.2%)	72 (0.3%)	63 (0.1%)
Suicide and self-inflicted injuries by jumping from high place - Other man-made structures (E957.1)	134 (0.2%)	86 (0.3%)	48 (0.1%)
Suicide and self-inflicted injuries by jumping from high place - Natural sites (E957.2)	17 (0.02%)	DS	DS
Suicide and self-inflicted injuries by jumping from high place - Unspecified (E957.9)	21 (0.03%)	DS	DS
Suicide and self-inflicted injury by Jumping or lying before moving object (E958.0)	121 (0.2%)	64 (0.2%)	57 (0.1%)
Suicide and self-inflicted injury by Burns, fire (E958.1)	243 (0.3%)	102 (0.4%)	141 (0.3%)
Suicide and self-inflicted injury by Scald (E958.2)	DS	DS	DS
Suicide and self-inflicted injury by Extremes of cold (E958.3)	19 (0.02%)	19 (0.07%)	-
Suicide and self-inflicted injury by Electrocution (E958.4)	DS	DS	DS
Suicide and self-inflicted injury by Crashing of motor vehicle (E958.5)	105 (0.1%)	57 (0.2%)	43 (0.1%)
Suicide and self-inflicted injury by Crashing of aircraft (E958.6)	DS	DS	DS
Suicide and self-inflicted injury by Caustic substances, except poisoning (E958.7)	20 (0.03%)	DS	DS
Suicide and self-inflicted injury by Other specified means (E958.8)	5368 (6.9%)	3599 (13.8%)	1768 (3.4%)
Suicide and self-inflicted injury by Unspecified means (E958.9)	1899 (2.4%)	901 (3.5%)	997 (1.9%)
Late effects of self-inflicted injury (E959)	93 (0.1%)	80 (0.3%)	13 (0.02%)

DS = “Discharge Information Suppressed” since cell counts were less than or equal to 10 (As per data user agreement with AHRQ).

Note: Individual cell counts may not add to the global total since a single discharge may have more than one E-code in the dataset.

## Discussion

The current study examines a nationally representative sample of hospital based emergency departments to provide estimates of ED visits attributed to attempted suicides and self inflicted injuries among children ages 18 years and younger in the United States. The results suggest that a greater proportion of such visits occurred among females (66.4% of the total visits). The frequently reported sources of injuries were attempts to poison themselves. Prior nationwide estimates also suggest that self poisoning is the most common method of self inflicted injuries and attempts to suicide [4]. However, this may be contributed by the higher percentage of female population shown in our study. A recent study by Branco et al. demonstrated a distinct sex differences in the mechanism of self-inflicted injuries in childhood and adolescents (ages 18 years and younger). Among male adolescents, shooting was found to be the most common mechanism, while poisoning was the dominant mechanism in female victims [8]. Fortunately, majority of the incidents tend to be non-fatal among children. Previous studies have shown that use of firearms is the leading cause of fatal self inflicted injuries [9]–[11]. In the current study, use of firearms was reported in less than 1% of all ED visits and this could explain the low mortality rates observed. Restricting access to firearms has been reported to be an effective approach to prevent suicides [9], [10]. The economics associated with treating children with these injuries in ED and hospital settings are also presented and highlight the public health burden of the problem.

The majority of patients in our study were discharged routinely (46.4% of the total visits), suggesting that a large group of children visiting the ED due to self-inflicted injuries and attempted suicides may have adequate and safe support system. Discharge home is usually considered for those adolescents who are not actively suicidal and who have an individual that can closely monitor their behaviors [12]. However, we do not know whether these group of discharged children received proper follow-up psychiatric and psychosocial therapies that are tailored to each particular suicidal ideation. Consequently, the importance of personal approach in addressing the issue and accompanying the patient to overcome such ideation throughout should be reinforced among the health care providers, as suggested by Polewka et al. [13].

The results presented in the current study are subject to several limitations which arise from using secondary hospital discharge datasets. We looked at external cause of injury codes to identify possible attempts to suicide and self inflicted injuries among children. The actual reason or cause for an ED visit cannot be identified using the current dataset. The estimates presented in the current study are not representative of all attempts to suicide or self inflicted injuries among children in the United States as the current study examines only those that visit an ED. Injuries that are treated in non-hospital based clinics are not presented in the current study. Emergency department charges presented in the current study are not available for close to 21% of all ED visits in the NEDS database. Consequently the economic burden in terms of emergency department charges for the entire United States is an underestimation of the true economic impact. Costs associated with treating these injuries outside the selected hospital settings are not captured in the NEDS dataset and thus not presented in the current study. Considering that we used external cause of injury codes to select cases, any coding inconsistencies during the data collection stage will introduce errors. Circumstances leading to injuries reported in the current study are not available and consequently these results cannot be used to tailor preventive programs to high-risk individuals. However, if an ideation of utilizing certain methods of self-inflicted injuries is suspected or identified in children at an earlier stage, our study does indicate that a particular cohort of children may be at a higher risk for future suicidal attempts that will most likely result in ED visits. In addition, the degree and extent of injuries by methods described in Table 2 cannot be determined and thus, whether the patient was hospitalized following the ED visit due to physically compromised state or mentally compromised state cannot be discerned. Finally, it should be kept in perspective that the unit of analysis is each individual visit to an ED and not the individual patient. There is no unique identifier to track individual patients in the NEDS dataset. This precludes us from examining previous hospitalizations due to intentional or unintentional injuries. It has been shown that there is a strong association between previous hospitalizations for injury and youth suicide [14].

### Conclusions

A total of 77,420 ED visits among children ages 18 years and younger were attributed to attempted suicides and self-inflicted injuries in the United States during the year 2007. 227 children died either in the ED’s or in hospitals. The current study results highlight the national burden associated with such injuries among children and demonstrate that there still exists a myriad of children who are at risk for suicides in the United States.
